# Plitidepsin in adult patients with COVID-19 requiring hospital admission: A long-term follow-up analysis

**DOI:** 10.3389/fcimb.2023.1097809

**Published:** 2023-02-22

**Authors:** Jose F. Varona, Pedro Landete, Roger Paredes, Roberto Vates, Miguel Torralba, Pablo Guisado-Vasco, Lourdes Porras, Patricia Muñoz, Paloma Gijon, Julio Ancochea, Elena Saiz, Fernanda Meira, Jose M. Jimeno, Jose A. Lopez-Martin, Vicente Estrada

**Affiliations:** ^1^ Departamento de Medicina Interna, Hospital Universitario HM Monteprincipe, HM Hospitales, Madrid, Spain; ^2^ Facultad de Medicina, Universidad San Pablo-Centro de Estudios Universitarios (CEU), Madrid, Spain; ^3^ Departamento de Neumología, Hospital Universitario La Princesa, Madrid, Spain; ^4^ Facultad de Medicina, Universidad Autónoma de Madrid, Madrid, Spain; ^5^ Infectious Diseases Department, IrsiCaixa Acquired Immunodeficiency Syndrome (AIDS) Research Institute, Barcelona, Spain; ^6^ Servicio de Enfermedades Infecciosas Hospital Germans Trias I Pujol, Barcelona, Spain; ^7^ Internal Medicine Department, Hospital Universitario de Getafe, Madrid, Spain; ^8^ Medicine Department, Health Sciences Faculty, University of Alcalá, Madrid, Spain; ^9^ Internal Medicine Department, Guadalajara University Hospital, Guadalajara, Spain; ^10^ Internal Medicine Department, Hospital Universitario Quironsalud Madrid, Madrid, Spain; ^11^ Departamento de Medicina, Facultad de Ciencias Biomédicas y de la Salud, Universidad Europea, Madrid, Spain; ^12^ Internal Medicine, Hospital General de Ciudad Real, Ciudad Real, Spain; ^13^ Clinical Microbiology and Infectious Diseases Department, Instituto de Investigación Sanitaria Gregorio Marañón (IiSGM), Hospital General Universitario Gregorio Marañón, Madrid, Spain; ^14^ Virology Unit, PharmaMar, SA, Madrid, Spain; ^15^ Departamento de Medicina Interna Hospital Clínico San Carlos, Madrid, Spain; ^16^ Facultad de Medicina, Universidad Complutense de Madrid, Madrid, Spain

**Keywords:** plitidepsin, COVID-19, SARS-CoV-2, long COVID, post-COVID-19 complications

## Abstract

**Introduction:**

The APLICOV-PC study assessed the safety and preliminary efficacy of plitidepsin in hospitalized adult patients with COVID-19. In this follow-up study (E-APLICOV), the incidence of post-COVID-19 morbidity was evaluated and any long-term complications were characterized.

**Methods:**

Between January 18 and March 16, 2022, 34 of the 45 adult patients who received therapy with plitidepsin in the APLICOV-PC study were enrolled in E-APLICOV (median time from plitidepsin first dose to E-APLICOV enrollment, 16.8 months [range, 15.2–19.5 months]). All patients were functionally autonomous with regard to daily living (Barthel index: 100) and had normal physical examinations.

**Results:**

From the APLICOV-PC date of discharge to the date of the extension visit, neither Common Terminology Criteria for Adverse Events version 5.0 (CTCAE v5) grade 3-4 complications nor QT prolongation or significant electrocardiogram (EKG) abnormalities were reported. Five (14.7%) patients had another COVID-19 episode after initial discharge from APLICOV-PC, and in 2 patients (5.9%), previously unreported chest X-ray findings were documented. Spirometry and lung-diffusion tests were normal in 29 (85.3%) and 27 (79.4%) patients, respectively, and 3 patients needed additional oxygen supplementation after initial hospital discharge. None of these patients required subsequent hospital readmission for disease-related complications.

**Discussion:**

In conclusion, plitidepsin has demonstrated a favorable long-term safety profile in adult patients hospitalized for COVID-19. With the constraints of a low sample size and a lack of control, the rate of post-COVID-19 complications after treatment with plitidepsin is in the low range of published reports. (ClinicalTrials.gov Identifier: NCT05121740; https://clinicaltrials.gov/ct2/show/NCT05121740).

## Introduction

As of 2 October 2022, the severe acute respiratory syndrome coronavirus 2 (SARS-CoV-2) pandemic has resulted in more than 615 million infections and 6.5 million deaths, at significant costs to healthcare systems and societies worldwide (World Health Organization, 2022). Though initial public health responses focused on reducing the acute burden of coronavirus disease 2019 (COVID-19), it has become increasingly apparent that SARS-CoV-2 infection can also provoke longer-term mental and physical health consequences, thus heightening the concern of the healthcare systems (Greenhalgh et al., 2020; National Institute for Health and Care Research, 2020; National Institute for Health and Care Excellence, 2020). The persistence of symptoms –such as fatigue, dyspnea, chest pain, cognitive disturbances, or arthralgia – 3 months after SARS-CoV-2 infection is referred to as post-COVID-19 disease or “long COVID” (National Institute for Health and Care Excellence, 2020). Between 10 and 25% of patients will experience long COVID, resulting in a significant limitation of daily activities, an increase in long-term sick leave from work, and the appearance of sequelae that may continue for more than one year (Carfì et al., 2020; Augustin et al., 2021).

Post-COVID-19 sequelae in patients who required hospitalization due to a severe SARS-CoV-2 infection are generally a consequence of structural damage to different organs by the infection itself and/or associated complications. Various studies point to sequelae not only restricting the respiratory apparatus but also affecting the cardiovascular system, the kidneys and the central and peripheral nervous system (Carfì et al., 2020; Salehi et al., 2020; Vindegaard and Benros, 2020; Ojha et al., 2020; Sudre et al., 2021; Huang et al., 2021). Psychiatric and psychological sequelae have also been documented (Vindegaard and Benros, 2020).

Risk factors for post-COVID-19 sequelae include the severity of acute COVID-19 infection, age, biological sex and sex hormones, and the presence of pre-existing conditions (Koc et al., 2022). Furthermore, it has been reported that viral load during acute COVID-19 may correlate with the severity of long COVID manifestation, and a rapid drop in the viral load of patients with acute COVID-19 infection may be protective from long COVID responses (Koc et al., 2022). Treatments that facilitate rapid resolution of the acute infection could therefore be protective against the effects of long COVID.

The APLICOV-PC proof-of-concept study (NCT04382066) assessed the safety and preliminary efficacy of 3 dose levels of plitidepsin (1.5 mg, 2.0 mg and 2.5 mg) administered for three consecutive days in hospitalized adult patients with COVID-19 (Varona et al., 2022). The trial met the primary endpoint of safety and feasibility of the 3 plitidepsin doses administered. In addition, results from APLICOV-PC suggested that plitidepsin was associated with reductions of viral load (mean 3.25-log_10_ reduction in baseline viral load by Day 15), inducing recovery (87% of the patients had moderate to severe illness when they entered the study; by Day 15, 82% of the patients had been discharged), and providing relevant impact on lymphocyte reconstitution and other inflammatory parameters (Varona et al., 2022).

The E-APLICOV study (NCT05121740) described herein was designed to evaluate whether plitidepsin treatment in patients hospitalized for COVID-19 could have a relevant impact on the emergence of long-term sequelae resulting from SARS-CoV-2 infection.

## Methods

### Study design and participants

APLICOV-PC was conducted in 10 hospital centers in Spain between May 12, 2020 and November 26, 2020 (second COVID-19 pandemic wave). Details and results of the study have been previously published (Varona et al., 2022). This multi-site extension of the APLICOV-PC clinical study (ran from January 18, 2022 to March 16, 2022) invited patients who received previous therapy with plitidepsin for COVID-19. Patients were enrolled after signing informed consent to participate in this extension study. The patients included in the extension study received no treatment whatsoever in relation to the study.

### Procedures

The sites participating in the study were supervised by monitors appointed by the sponsor. Monitors performed periodic visits to the site before, during and at the end of the trial, or failing that, by telephone contact or written communication. Remote verification of source data were performed in agreement with the participating centers.

Data were collected through electronic case report forms (eCRFs) from each center participating in the study, and were provided by the Clinical Research Organization (CRO). The data was collected and processed with the appropriate precautions to guarantee confidentiality and compliance with the current legislation regarding data privacy (EU Regulation 2016/679 of the European Parliament and of the Council of 27 April 2016 on the protection of natural persons with regard to the processing of personal data and on the free movement of such data and with Organic Law 03/2018 on protection of personal data and guarantee of digital rights).

### Study objective

The main objective of this study was to evaluate the incidence of post-COVID-19 morbidity and characterize the profile of complications in patients who participated in the APLICOV-PC study, assessing the incidence of post-COVID-19 complications after exposure to therapeutic intervention with plitidepsin at flat doses of 1.5, 2.0, and 2.5 mg/day, for 3 consecutive days as a 90-min IV infusion (Varona et al., 2022).

To complete this objective, the following information was gathered for the time spanning from the patient’s last visit in the APLICOV-PC study through the end of the extension study: 1) readmissions to hospitals and their causes; 2) the need for oxygen therapy and duration of the same; and 3) the incidence of complications (See **Supplementary Table 1**).

### Statistical analysis

All data has been analyzed with the SAS statistical analysis system, version 9.4. All analyses have been carried out mainly by descriptive statistical methods. Continuous endpoints have been described using maximum, minimum, Q1 and Q3, mean, median and standard deviation. Categorical endpoints have been described with frequencies and percentages as well as the exact 95% confidence interval of the relevant study variables. All demographic and patient characteristics are reported using the initiation of the E-APLICOV study as baseline unless otherwise noted.

## Results

**Figure 1:**
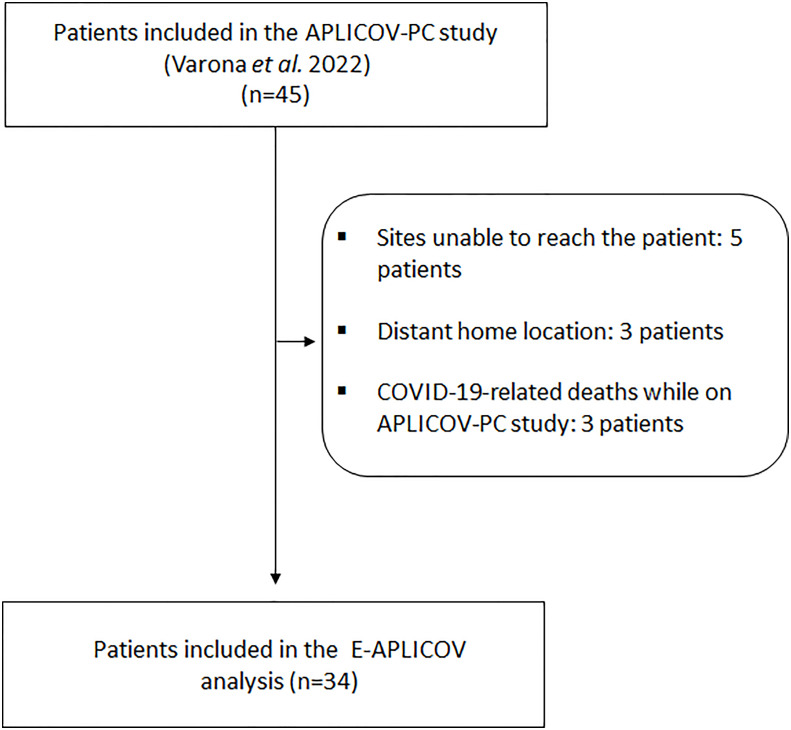
Flowchart of exclusions.

Of the 45 adult patients who received therapy with plitidepsin in the APLICOV-PC study, 34 were enrolled into this extension (**Figure 1**). Of those included in E-APLICOV, 5 (14.7%), 19 (55.9%) and 10 (29.4%) patients had mild, moderate, and severe COVID-19 (FDA criteria), respectively, at the time of treatment with plitidepsin (**Table 1**). A median of 16.8 months (range 15.2 – 19.5 months) had elapsed from the time patients received their first dose of plitidepsin to the start of this extension (**Table 1**). Most patients were male (23 patients, 67.6%) and Caucasian (23 patients, 67.6%) (**Table 1**). Their median age was 53 years (range: 33-72 years) and their median body-mass index (BMI) was 29.7 kg/m^2^ (range: 18.4-46.2 kg/m^2^) (**Table 1**). Obesity (BMI > 30 kg/m^2^) was observed in 16 (47%) patients. Sixteen (47.1%) patients had two or more co-morbidities at randomization (**Table 1**). Eighty-five percent of patients had received some form of pharmacological treatment at E-APLICOV study entry. Thirty-eight percent of all patients were treated with lipid modifying agents and 35.3% with drugs used in diabetes. Analgesics (23.5% of patients) and anti-inflammatory and antirheumatic products (23.5% of patients) were also among the most frequently used medications for patients included in the study (See **Supplementary Table 2**). There was only 1 (2.9%) unvaccinated patient at the start of E-APLICOV, whereas 6 (17.6%), 21 (61.8%), and 6 (17.6%) patients, respectively, had received 1, 2, and 3 doses of a COVID-19 vaccine since the conclusion of the initial study (**Table 1**). A total of 3 patients were seronegative (8.8%) at entry into the extension study (**Table 1**). The median values of analytical parameters relevant to COVID-19 at study entry are shown in **Supplementary Table 3**. Patients in this analysis had a median total lymphocyte count of 1.98 x10^9^ cells/L (interquartile range [IQR], 1.56–2.56), C-reactive protein [CRP] of 1.36 mg/dL (IQR, 0.9–2.9) and D-dimer of 240.5 ng/mL (IQR, 201–343).

**Table 1:** Demographic and baseline characteristics for the 34 patients included in the E-APLICOV study.

	1.5 mg N=9	2.0 mg N=14	2.5 mg N=11	Total N=34
**Age, median (range) years**	53 (33-70)	52,5 (36-72)	54 (43-68)	53 (33-72)
**Gender, n (%) male**	7 (77.8)	10 (71.4)	6 (54.5)	23 (67.6)
Ethnicity, n (%)				
**Caucasian**	7 (77.8)	9 (64.3)	7 (63.6)	23 (67.6)
**Latin**	2 (22.2)	3 (21.4)	4 (36.4)	9 (26.5)
**North African**	–	1 (7.1)	–	1 (2.9)
**Asian**	–	1 (7.1)	–	1 (2.9)
**Body mass index, median (range) kg/m^2^**	27.7 (18.4-34.2)	30.3 (22.0-46.2)	29.8 (21.9-41.5)	29.7 (18.4-46.2)
**Median (range) time from plitidepsin first dose to E-APLICOV inclusion, months**	17.6 (15.3-19.5)	17.2 (15.7-18.6)	16.2 (15.2-17.8)	16.8 (15.2-19.5)
Number of comorbidities, n (%)				
**None**	3 (33.3)	2 (14.3)	0 (0)	5 (14.7)
**One**	1 (11.1)	6 (42.9)	6 (54.5)	13 (38.2)
**Two or more**	5 (55.6)	6 (42.9)	5 (45.5)	16 (47.1)
Disease status at randomization, n (%) ^A^				
**Mild COVID-19**	1 (11.1)	3 (21.4)	1 (9.1)	5 (14.7)
**Moderate COVID-19**	5 (55.6)	7 (50)	7 (63.6)	19 (55.9)
**Severe COVID-19**	3 (33.3)	4 (28.6)	3 (27.3)	10 (29.4)
Vaccination status (COVID-19)				
**Non-vaccinated patients, n (%)**	1 (11.1)	–	–	1 (2.9)
**Vaccinated patients, n (%)**	8 (88.9)	14 (100)	11 (100)	33 (97.1)
**1 dose**	1 (11.1)	4 (28.6)	1 (9.1)	6 (17.6)
**2 doses**	4 (44.4)	7 (50.0)	10 (90.9)	21 (61.8)
**3 doses**	3 (33.3)	3 (21.4)	–	6 (17.6)
Serostatus against SARS-CoV-2, n (%)				
**Negative**	1 (11.1)	1 (7.1)	1 (9.1)	3 (8.8)
**Positive**	8 (88.9)	13 (92.9)	10 (90.9)	31 (91.2)

^A^ Estimated using baseline assessments recorded in Electronic Data Capture using severity definitions in FDA Guidance for Industry “COVID-19: Developing Drugs and Biological Products for Treatment or Prevention”: mild (no shortness of breath or dyspnea, SpO_2_ ≥ 95%, respiratory rate < 20 breaths/min, heart rate < 90 bpm), moderate (shortness of breath with exertion, SpO_2_ > 93% but < 95%, respiratory rate ≥ 20 breaths/min or heart rate ≥ 90 bpm), and severe (shortness of breath at rest or respiratory distress, SpO_2_ ≤ 93%, respiratory rate ≥ 30 breaths/min, heart rate ≥ 125 bpm).

bpm, beats per minute; FDA, Food and Drug Administration; Kg, kilogram; m^2^, square meter; mg, milligram; min, minutes; N, n, number; SpO_2_, oxygen saturation. –, No patients.

At study entry, none of the patients showed functional limitations for their daily living activities (Barthel index: 100) and there were no reports of physical and neurological abnormalities. There were no CTCAE v5 grade 3–4 complications from the date of APLICOV-PC discharge to the date of the extension visit. Nearly 15% of patients (n=5) were diagnosed with COVID-19 after the completion of the APLICOV-PC study (approximately 13-15 months after the last patient inclusion in the APLICOV-PC study). Among the post-COVID-19 complications occurring in the patients analyzed in the study (See **Supplementary Tables 4**, **5** **)**, the investigators found the following events, reported as grade 2 disease-related complications, in one patient each (2.9%): alopecia, cough, diabetic metabolic decompensation, hyperglycemia, and interstitial lung disease. Grade 2 asthenia was described in 2 patients (5.9%) (**Table 2**). There were no reports on clinically relevant abnormalities in hematological parameters. None of the patients experienced a thromboembolic event.

**Table 2:** Summary of post-COVID-19 complications, related to study disease, overall. Worst grade per patient.

Post-COVID-19 complications	Grade						Total	
	Missing		G1		G2			
	N	%	N	%	N	%	N	%
**Alanine aminotransferase increased**	.	.	1	2.9	.	.	1	2.9
**Alopecia**	.	.	.	.	1	2.9	1	2.9
**Anxiety**	.	.	2	5.9	.	.	2	5.9
**Aspartate aminotransferase increased**	.	.	1	2.9	.	.	1	2.9
**Asthenia**	.	.	11	32.4	2	5.9	13	38.2
**Attention deficit**	.	.	5	14.7	.	.	5	14.7
**Back pain**	.	.	1	2.9	.	.	1	2.9
**Blood cholesterol increased**	.	.	1	2.9	.	.	1	2.9
**Blood triglycerides increased**	.	.	1	2.9	.	.	1	2.9
**COVID-19 pneumonia**	.	.	1	2.9	.	.	1	2.9
**Chest pressure**	.	.	1	2.9	.	.	1	2.9
**Cough**	.	.	4	11.8	1	2.9	5	14.7
**Diabetic metabolic decompensation**	.	.	.	.	1	2.9	1	2.9
**Diarrhea**	.	.	1	2.9	.	.	1	2.9
**Dizziness**	1	2.9	3	8.8	.	.	4	11.8
**Dyspnea**	.	.	1	2.9	.	.	1	2.9
**Febricula**	.	.	1	2.9	.	.	1	2.9
**Fibrin D-dimer increased**	.	.	1	2.9	.	.	1	2.9
**Gamma-glutamyltransferase increased**	.	.	2	5.9	.	.	2	5.9
**General malaise**	.	.	5	14.7	.	.	5	14.7
**Headaches**	.	.	3	8.8	.	.	3	8.8
**Hyperglycemia**	.	.	.	.	1	2.9	1	2.9
**Interstitial lung disease**	.	.	.	.	1	2.9	1	2.9
**Joint pains**	.	.	2	5.9	.	.	2	5.9
**Low mood**	.	.	3	8.8	.	.	3	8.8
**Memory lapses**	.	.	4	11.8	.	.	4	11.8
**Muscle aches and pains**	.	.	3	8.8	.	.	3	8.8
**Palpitations**	.	.	2	5.9	.	.	2	5.9
**Serum ferritin increased**	.	.	2	5.9	.	.	2	5.9
**Tingling in extremity**	.	.	5	14.7	.	.	5	14.7

G, grade; N, number. –, No patients.

Two patients (5.9%) presented chest X-ray findings (hilar adenopathies and bilateral micronodules, impingement of the left lateral costophrenic sinus and calcified atheromatosis of the aortic arch) not reported previously. Electrocardiogram (EKG) abnormalities (e.g., atrial fibrilation, left axis deviation or sinus tachycardia) were found in 5 patients (14.7%) but were not judged as clinically significant. No QT prolongation was described in any of the 34 patients.

Spirometry and lung-diffusion tests were normal in 29 (85.3%) and 27 (79.4%) patients, respectively, at study entry (**Table 3**). The pre-test 6-minute walk test scored 0–2 (no or slight dyspnea) in all patients (Borg scale). After the test, 24 (70.6%) patients had scores of 0–2, 6 (17.6%) patients of 2.5–4 (moderate dyspnea), and 4 (11.8%) patients had severe dyspnea (**Supplementary Table 6**). Only 2 patients, 1 with moderate COVID-19 and 1 in the severe group, stopped or paused during the test and none of the patients experienced symptoms at the end of the exercise.

**Table 3:** Pulmonary function assessment.

Pulmonary function assessment			1.5 mg		2 mg		2.5 mg		Total	
			N	%	N	%	N	%	N	%
**Spirometry**	**FVC result**	**Normal**	7	77.8	14	100.0	11	100.0	32	94.1
		**Reduced**	2	22.2	.	.	.	.	2	5.9
	**FEV_1_ result**	**Normal**	7	77.8	13	92.9	9	81.8	29	85.3
		**Reduced**	2	22.2	1	7.1	2	18.2	5	14.7
	**FEV_1_/FVC result**	**Normal**	9	100.0	12	85.7	8	72.7	29	85.3
		**Reduced**	.	.	2	14.3	3	27.3	5	14.7
**Dyspnea assessment**	**mMRC**	**0**	5	55.6	7	50.0	9	81.8	21	61.8
		**1**	2	22.2	6	42.9	2	18.2	10	29.4
		**2**	.	.	1	7.1	.	.	1	2.9
		**3**	2	22.2	.	.	.	.	2	5.9
**Lung-diffusion testing**	**DLCO result**	**Normal**	9	100.0	10	71.4	8	72.7	27	79.4
		**High**	.	.	3	21.4	2	18.2	5	14.7
		**Low**	.	.	1	7.1	1	9.1	2	5.9

0 (Symptoms: Little; Description: Dyspnea only with strenuous exercise); 1 (Symptoms: Mild; Description: Dyspnea when hurrying or walking up a slight hill); 2 (Symptoms: Moderate; Description: Walks slower than people of the same age because of dyspnea or has to stop for breath when walking at own pace); 3 (Symptoms: Many; Description: Stops for breath after walking just under 100 m (100 yards) or after a few minutes); 4 (Symptoms: Very much; Description: Too dyspneic to leave house or breathless when dressing) DLCO, diffusing capacity of the lungs for carbon monoxide; FEV_1_, forced expiratory volume in one second; FVC, forced vital capacity; mg, milligram; mMRC, modified Medical Research Council; N, number. ., No patients.

Subsequent hospital readmission for disease-related complications was not required for any of the patients. Three patients (8.82%, 2 with severe and 1 with moderate COVID-19 disease) required additional oxygen supplementation, *via* nasal cannula or Venturi mask, after initial hospital discharge.

## Discussion

Treatments to manage or prevent the development of sequelae arising from acute SARS-CoV-2 infection are urgently needed. Growing evidence suggests that the pathophysiologic model underlying post-COVID-19 sequelae stems from a dysregulated immune system that, after acute infection, continues releasing aberrantly high levels of proinflammatory cytokines that lead to chronic low-grade inflammation and multiorgan symptomatology (Buonsenso et al., 2022). Further, the hypothesis that a rapid reduction in SARS-CoV-2 viral load during acute infection may reduce the risk of post-COVID-19 complications is increasingly gaining strength (Rajan et al., 2021).

The previous APLICOV-PC proof-of-concept study confirmed plitidepsin safety in adult COVID-19 patients requiring hospital admission. Data gathered suggested that treatment of hospitalized patients with plitidepsin could sharply reduce SARS-CoV-2 viral load, promote recovery, and positively impact on the absolute lymphocyte counts and other inflammatory parameters (Varona et al., 2022). This trend toward increasing the total number of lymphocytes is of utmost importance given that the depth of lymphopenia may be associated with poor prognosis, including higher COVID-19 mortality (Lee et al., 2021). To the best of our knowledge, an increase in lymphocyte counts has not been reported with other antiviral therapies (Barratt-Due et al., 2021).

Due to plitidepsin’s putative effect on rapidly reducing the SARS-CoV-2 viral load and normalizing certain immune system parameters, we sought to characterize the incidence of post-COVID-19 morbidity and long-term complications in patients who participated in APLICOV-PC. As the last patient in the APLICOV-PC study was enrolled in November 2020, there was already an extensive margin of follow-up time of almost 12 months to evaluate these parameters in this study. Despite the relatively high severity of COVID-19 (85% were graded as moderate to severe in the original study) and prevalence of comorbidity (85% of patients had ≥1 comorbidity), we observed few long-term complications in this group of patients previously treated with plitidepsin. None of the patients included in the E-APLICOV study had experienced functional restrictions in performing physical actions needed in everyday life. Reports of neurological Grade 1 post-COVID-19 complications, deemed to be related to study disease, included attention deficit (n=5; 14.7%), memory lapses (n=4; 11.8%), low mood (n=3; 8.8%), headaches (n=3; 8.8%), and anxiety (n=2; 5.9%). These low rates are in contrast with reports from an electronic follow-up record of 236,379 patients during the first six months following COVID-19 diagnosis, in which neuropsychiatric complications emerged in 34% of cases, not including headache. The most commonly reported complications include mood and anxiety disorders and psychoses (24%), neuropathies (2.1%), and dementia (0.67%) (Taquet et al., 2021). On the other hand, the most frequent neurological symptoms are headache and cognitive changes, described in up to 68% and 81% of patients, respectively, with some sort of neurological symptoms after week 12 following acute infection (Graham et al., 2021).

In patients who present with severe COVID-19, the main sequelae is the development of pulmonary fibrosis. According to a recent meta-analysis, approximately 30% of patients hospitalized with pneumonia due to SARS-CoV-2 have shown fibrotic changes that persist for the first 12 months after discharge from the hospital (Fabbri et al., 2022). In this study, grade 1 COVID-19 pneumonia was reported in only 1 patient (2.9%) and only 2 patients (5.9%) presented chest X-ray findings, which were not suggestive of pulmonary fibrosis. None of the patients experienced a pulmonary embolism.

Finally, it should be highlighted that the dose range proposed in the APLICOV-PC study, which was based on pharmacokinetic/pharmacodynamic modeling, anticipated that antiviral concentrations of plitidepsin would be reached in distal anatomical compartments. *In-vivo* biodistribution of plitidepsin confirms that key organs in SARS-CoV-2 are exposed to therapeutic concentrations. Considering that SARS-CoV-2 infection is a systemic process that goes beyond affecting the respiratory tract, the biodistribution of plitidepsin might result in a widespread reduction of organ dysfunction, potentially protecting SARS-CoV-2-affected organs (Machhi et al., 2020; Rajan et al., 2021). Indeed, data from the COVERSCAN study conducted in the United Kingdom, which carried out serial Magnetic Resonance Imaging (MRI) scanning in a sample of 201 generally-healthy, middle-aged individuals, with COVID-19, showed evidence of mild organ impairment of the heart (32%), lungs (33%), kidneys (12%), liver (10%), pancreas (17%), and spleen (6%) (Rajan et al., 2021). Significant heart injuries, including myocarditis with reduced systolic function and arrhythmias, have been documented in patients with severe forms of COVID-19. Myocardial injury has been reported, which may be due to direct damage to the cardiomyocytes, systemic inflammation, myocardial interstitial fibrosis, and hypoxia (Babapoor-Farrokhran et al., 2020). Due to significant myocardial injuries in patients with severe COVID-19 symptoms, the morbidity and lethality of the illness could be high (Aggarwal et al., 2020; Clerkin et al., 2020; Bansal, 2020). Moreover, it has been shown that right ventricular abnormalities can occur after SARS-CoV-2 infection and likely reflect the consequences of COVID-19-associated severe pneumonia (Singh et al., 2022). It should be noted, however, that no clinically significant functional heart abnormalities were found in patients of the E-APLICOV study. At the E-APLICOV study entry, spirometry and lung-diffusion tests were abnormal in only 14.7% and 20.6% of the patients, respectively, percentages below the COVID-19-derived lung dysfunction rates previously described in the literature (Rajan et al., 2021).

In summary, considering the absence of CTCAE v5 grade 3-4 complications and of clinically significant EKG abnormalities, as well as the low rate of disease-related complications, chest X-ray findings and requirement of oxygen supplementation in patients included in this study, plitidepsin has demonstrated long-term safety in adult patients hospitalized for COVID-19. Despite the limitations of a low sample size and a lack of control group in APLICOV-PC, the rate of post-COVID-19 complications after treatment with plitidepsin appears to be in the lower limit of the 95% confidence interval of the prevalence of post-COVID-19 complications reported in a recent meta-analysis [80% (95% CI 65–92)] (Lopez-Leon et al., 2021).

## Data availability statement

The original contributions presented in the study are included in the article/**Supplementary Material**. Further inquiries can be directed to the corresponding author.

## Ethics statement

The studies involving human participants were reviewed and approved by CEIm HM Hospitales. The patients/participants provided their written informed consent to participate in this study.

## Author contributions

The specific additional participation of each author is as follows: JV: conceptualization, investigation, writing and reviewing of the original draft. PL: investigation. RP: conceptualization and investigation. RV, MT, PG-V, LP, PM, PG, and JA: Investigation. ES and FM: data supervision. JJ: conceptualization, formal analysis, supervision, validation, investigation, methodology, and writing & critical review of the article. JL-M: conceptualization, data curation, software, formal analysis, supervision, validation, investigation, visualization, methodology, writing & critical review of the article. VE: conceptualization and investigation. All authors contributed to the article and approved the submitted version.
