# Supplementary material for: Plitidepsin in adult patients with COVID-19 requiring hospital admission: A long-term follow-up analysis
Source: Front Cell Infect Microbiol. 2023 Feb 22;13:1097809. doi: 10.3389/fcimb.2023.1097809 (PMC9992643; doi:10.3389/fcimb.2023.1097809)
Supplement: Supplementary file 1 [file DataSheet_1.pdf]

Supplementary Material

Plitidepsin in Adult Patients with COVID-19 Requiring Hospital Admission: A Long-term Follow-up Analysis

Jose F Varona[1,2*], Pedro Landete[3,4], Roger Paredes[5,6], Roberto Vates[7], Miguel Torralba[8,9], Pablo Guisado-Vasco[10,11], Lourdes Porras[12], Patricia Muñoz[13], Paloma Gijon[13], Julio Ancochea[3,4], Elena Saiz[14], Fernanda Meira[14], Jose M Jimeno[14], Jose A Lopez-Martin[14], Vicente Estrada[15,16]

[1]Departamento de Medicina Interna, Hospital Universitario HM Monteprincipe, HM Hospitales, Madrid, Spain jfvarona@hmhospitales.com.
[2]Facultad de Medicina, Universidad San Pablo-CEU, Madrid, Spain.
[3]Departamento de Neumología, Hospital Universitario La Princesa, Madrid, Spain.
[4]Facultad de Medicina, Universidad Autónoma de Madrid, Madrid, Spain.
[5]Infectious Diseases Department, IrsiCaixa AIDS Research Institute, Barcelona, Spain.
[6]Servicio de Enfermedades Infecciosas, Hospital Germans Trias I Pujol, Badalona, Spain.
[7]Internal Medicine Department, Hospital Universitario de Getafe, Madrid, Spain.
[8]Medicine Department, Health Sciences Faculty, University of Alcalá, Madrid, Spain.
[9]Internal Medicine Department, Guadalajara University Hospital, Guadalajara, Spain.
[10]Internal Medicine Department, Hospital Universitario Quironsalud Madrid, Madrid, Spain.
[11]Departamento de Medicina, Facultad de Ciencias Biomédicas y de la Salud, Universidad Europea, Madrid, Spain.
[12]Internal Medicine, Hospital General de Ciudad Real, Ciudad Real, Spain.
[13]Clinical Microbiology and Infectious Diseases Department, Instituto de Investigación Sanitaria Gregorio Marañón (IiSGM), Hospital General Universitario Gregorio Marañón, Madrid, Spain.
[14]Virology Unit, PharmaMar, SA, Madrid, Spain
[15]Departamento de Medicina Interna, Hospital Clínico San Carlos, Madrid, Spain.
[16]Facultad de Medicina, Universidad Complutense de Madrid, Madrid, Spain.

*** Correspondence:**
Corresponding Author
Jose Felipe Varona Arche at jfvarona@hmhospitales.com

Keywords: Plitidepsin; COVID-19; SARS-CoV-2; long COVID; post-COVID-19 complications

Supplementary Tables

Supplementary Table 1. Prevalent post-COVID-19 complications previously reported in published analysis.

**List of COVID-19 complications**
Fatigue/asthenia
General malaise
Headaches
Low mood
Muscle aches and pains
Joint pains
Attention deficit
Chest pressure
Anxiety
Febricula
Cough
Memory lapses
Diarrhea
Palpitations
Dizziness
Tingling in extremity
Others (specify)

Supplementary Table 2. Pharmacological treatments at study entry

Pharmacological treatment		1.5 mg		2 mg		2.5 mg		Total	
		N	%	N	%	N	%	N	%
**Has the patient received any pharmacological treatment?**		7	77.8	12	85.7	10	90.9	29	85.3
**Alimentary tract and metabolism**	**Drugs for acid-related disorders**	2	22.2	3	21.4	1	9.1	6	17.6
	**Drugs used in diabetes**	2	22.2	6	42.9	4	36.4	12	35.3
	**Vitamins**	2	22.2	2	14.3	1	9.1	5	14.7
**Anti-infectives for systemic use**	**Antibacterials for systemic use**	1	11.1	1	7.1	.	.	2	5.9
	**Antivirals for systemic use**	.	.	.	.	1	9.1	1	2.9
**Blood and blood-forming organs**	**Antithrombotic agents**	3	33.3	3	21.4	.	.	6	17.6
	**Antianemic preparations**	.	.	1	7.1	.	.	1	2.9
**Cardiovascular system**	**Beta blocking agents**	1	11.1	1	7.1	.	.	2	5.9
	**Cardiac therapy**	1	11.1	1	7.1	.	.	2	5.9
	**Lipid modifying agents**	5	55.6	4	28.6	4	36.4	13	38.2
	**Agents acting on the renin-angiotensin system**	.	.	3	21.4	2	18.2	5	14.7
	**Antihypertensives**	.	.	1	7.1	.	.	1	2.9
	**Calcium channel blockers**	.	.	2	14.3	2	18.2	4	11.8
	**Diuretics**	.	.	2	14.3	.	.	2	5.9
**Dermatologicals**	**Antifungals for dermatological use**	1	11.1	1	7.1	.	.	2	5.9
**Musculo-skeletal system**	**Anti-inflammatory and antirheumatic products**	2	22.2	3	21.4	3	27.3	8	23.5
	**Drugs for treatment of bone diseases**	1	11.1	.	.	.	.	1	2.9
	**Muscle relaxants**	1	11.1	2	14.3	.	.	3	8.8
	**Antigout preparations**	1	11.1	2	14.3	1	9.1	4	11.8
**Nervous system**	**Analgesics**	2	22.2	4	28.6	2	18.2	8	23.5
	**Psychoanaleptics**	2	22.2	1	7.1	1	9.1	4	11.8

**Pharmacological treatment**		**1.5 mg**		**2 mg**		**2.5 mg**		**Total**	
		**N**	**%**	**N**	**%**	**N**	**%**	**N**	**%**
	**Psycoleptics**	1	11.1	1	7.1	.	.	2	5.9
	**Anti-Parkinson drugs**	.	.	1	7.1	.	.	1	2.9
**Respiratory system**	**Antihistamines for systemic use**	1	11.1	.	.	2	18.2	3	8.8
	**Drugs for obstructive airway diseases**	1	11.1	1	7.1	2	18.2	4	11.8
	**Nasal preparations**	.	.	1	7.1	.	.	1	2.9
**Systemic hormonal preparations, excluding sex hormones and insulins**	**Corticosteroids for systemic use**	2	22.2	2	14.3	.	.	4	11.8
	**Thyroid therapy**	1	11.1	2	14.3	1	9.1	4	11.8
**Genitourinary system and hormones**	**Urologicals**	.	.	2	14.3	2	18.2	4	11.8
	**Other gynecologicals**	.	.	.	.	1	9.1	1	2.9

Supplementary Table 3. Blood laboratory parameters at study entry

	**1.5 mg N=9**	**2.0 mg N=14**	**2.5 mg N=11**	**Total N=34**
**Leukocytes-10^9/L, median (IQR)**	6.67 (5.45-7.7)	6 (5.36-7.88)	7.06 (5.54-7.78)	6.35 (5.45-7.72)
**Lymphocytes-10^9/L, median (IQR)**	1.8 (1.51-2.3)	2 (1.63-2.56)	2.09 (1.56-2.6)	1.98 (1.56-2.56)
**Neutrophils - 10^9/L, median (IQR)**	3.93 (3.00-4.37)	3.36 (2.90-4.10)	3.92 (3.10-4.72)	3.72 (3.00-4.52)
**Hemoglobin - g/dL, median (IQR)**	14.40 (13.80-15.00)	14.95 (14.50-15.90)	14.00 (13.40-15.40)	14.60 (13.80-15.40)
**Platelets - 10^9/L, median (IQR)**	233 (188-337)	237 (172-278)	247 (215-316)	240.5 (182-309)
**Ferritin (ng/mL), median (IQR)**	153 (82-196)	183 (80.5-223)	173.3 (62.5-323.1)	172 (65-223)
**Creatinine (mg/dL), median (IQR)**	0.79 (0.74-1.00)	0.71 (0.65-0.89)	0.9 (0.66-0.98)	0.8 (0.66-0.93)
**CRP (mg/dL), median (IQR)**	1.10 (0.62-1.2)	1.79 (0.9-2.9)	1.9 (1.1-4.37)	1.36 (0.9-2.9)
**ALT (ULN), median (IQR)**	0.44 (0.39-0.57)	0.50 (0.45-0.78)	0.51 (0.41-0.67)	0.50 (0.41-0.67)
**AST (ULN), median (IQR)**	0.51 (0.46-0.52)	0.51 (0.45-0.78)	0.51 (0.48-0.59)	0.51 (0.46-0.61)
**LDH (ULN), median (IQR)**	0.73 (0.59-0.79)	0.78 (0.62-0.91)	0.94 (0.83-1.01)	0.82 (0.65-0.95)
**D-dimer (ng/mL), median (IQR)**	203 (156-247)	310 (215-408)	304 (200-500)	240.5 (201-343)

ALT: alanine aminotransferase; AST: aspartate aminotransferase; CRP: C-reactive protein; dL: decilitre; IQR: interquartile range; IU: international unit; L: liter; LDH: lactate dehydrogenase; mg: milligram; N: number; ng: nanogram; x ULN: normalized value respect to the Upper Limit of the Normal range.

Supplementary Table 4. Summary of post-COVID-19 complications, regardless of relationship, by dose cohort and overall. Worst per patient.

Post-COVID-19 complications - Regardless of relationship	1.5 mg		2 mg		2.5 mg		Total	
	N	%	N	%	N	%	N	%
Alanine aminotransferase increased	1	11.1	.	.	.	.	1	2.9
Aspartate aminotransferase increased	1	11.1	.	.	.	.	1	2.9
Asthenia	2	22.2	8	57.1	5	45.5	15	44.1
Attention deficit	2	22.2	4	28.6	4	36.4	10	29.4
Blood triglycerides increased	1	11.1	1	7.1	.	.	2	5.9
COVID-19 pneumonia	1	11.1	.	.	.	.	1	2.9
Cough	1	11.1	3	21.4	2	18.2	6	17.6
Diarrhea	1	11.1	2	14.3	1	9.1	4	11.8
Dizziness	1	11.1	2	14.3	2	18.2	5	14.7
Fibrin D dimer increased	1	11.1	.	.	.	.	1	2.9
Gamma-glutamyltransferase increased	1	11.1	1	7.1	.	.	2	5.9
Interstitial lung disease	1	11.1	.	.	.	.	1	2.9
Joint pains	1	11.1	3	21.4	4	36.4	8	23.5
Low density lipoprotein increased	1	11.1	.	.	.	.	1	2.9
Memory lapses	1	11.1	6	42.9	3	27.3	10	29.4
Muscle aches and pains	2	22.2	3	21.4	2	18.2	7	20.6
Palpitations	2	22.2	1	7.1	1	9.1	4	11.8
Serum ferritin increased	1	11.1	1	7.1	.	.	2	5.9
Tingling in extremity	4	44.4	6	42.9	1	9.1	11	32.4
Anxiety	.	.	5	35.7	.	.	5	14.7
Back pain	.	.	1	7.1	.	.	1	2.9
Blood cholesterol increased	.	.	1	7.1	.	.	1	2.9
Chest pressure	.	.	1	7.1	.	.	1	2.9

Post-COVID-19 complications - Regardless of relationship	1.5 mg		2 mg		2.5 mg		Total	
	N	%	N	%	N	%	N	%
**Chloasma**	.	.	1	7.1	.	.	1	2.9
**Epistaxis**	.	.	1	7.1	.	.	1	2.9
**Febricula**	.	.	1	7.1	.	.	1	2.9
**General malaise**	.	.	3	21.4	2	18.2	5	14.7
**Headaches**	.	.	2	14.3	2	18.2	4	11.8
**Libido decreased**	.	.	1	7.1	.	.	1	2.9
**Low mood**	.	.	5	35.7	.	.	5	14.7
**Male sexual dysfunction**	.	.	1	7.1	.	.	1	2.9
**Nasal congestion**	.	.	1	7.1	.	.	1	2.9
**Oropharyngeal pain**	.	.	1	7.1	.	.	1	2.9
**Tremor**	.	.	1	7.1	.	.	1	2.9
**Urinary tract infection**	.	.	1	7.1	.	.	1	2.9
**Alopecia**	.	.	.	.	1	9.1	1	2.9
**Diabetic metabolic decompensation**	.	.	.	.	1	9.1	1	2.9
**Dyspnea**	.	.	.	.	1	9.1	1	2.9
**Hyperglycaemia**	.	.	.	.	1	9.1	1	2.9
**Phlebitis**	.	.	.	.	1	9.1	1	2.9

mg: milligram; N: number.

Supplementary Table 5. Summary of post-COVID-19 complications, regardless of relationship, by severity of disease. Worst per patient.

Post-COVID-19 complications - Regardless of relationship	Severity							
	Mild		Moderate		Severe		Total	
	N	%	N	%	N	%	N	%
**Anxiety**	2	40.0	2	10.5	1	10.0	5	14.7
**Asthenia**	3	60.0	7	36.8	5	50.0	15	44.1
**Attention deficit**	2	40.0	6	31.6	2	20.0	10	29.4
**Chloasma**	1	20.0	.	.	.	.	1	2.9
**Diarrhea**	2	40.0	1	5.3	1	10.0	4	11.8
**General malaise**	1	20.0	3	15.8	1	10.0	5	14.7
**Joint pains**	2	40.0	4	21.1	2	20.0	8	23.5
**Low mood**	1	20.0	2	10.5	2	20.0	5	14.7
**Memory lapses**	2	40.0	6	31.6	2	20.0	10	29.4
**Muscle aches and pains**	2	40.0	3	15.8	2	20.0	7	20.6
**Oropharyngeal pain**	1	20.0	.	.	.	.	1	2.9
**Tingling in extremity**	3	60.0	5	26.3	3	30.0	11	32.4
**Alopecia**	.	.	1	5.3	.	.	1	2.9
**Back pain**	.	.	1	5.3	.	.	1	2.9
**Chest pressure**	.	.	1	5.3	.	.	1	2.9
**Cough**	.	.	4	21.1	2	20.0	6	17.6
**Diabetic metabolic decompensation**	.	.	1	5.3	.	.	1	2.9
**Dizziness**	.	.	2	10.5	3	30.0	5	14.7
**Dyspnea**	.	.	1	5.3	.	.	1	2.9
**Febricula**	.	.	1	5.3	.	.	1	2.9
**Headaches**	.	.	3	15.8	1	10.0	4	11.8
**Libido decreased**	.	.	1	5.3	.	.	1	2.9

Post-COVID-19 complications - Regardless of relationship	Severity							
	Mild		Moderate		Severe		Total	
	N	%	N	%	N	%	N	%
**Male sexual dysfunction**	.	.	1	5.3	.	.	1	2.9
**Palpitations**	.	.	1	5.3	3	30.0	4	11.8
**Phlebitis**	.	.	1	5.3	.	.	1	2.9
**Tremor**	.	.	1	5.3	.	.	1	2.9
**Urinary tract infection**	.	.	1	5.3	.	.	1	2.9
**Alanine aminotransferase increased**	.	.	.	.	1	10.0	1	2.9
**Aspartate aminotransferase increased**	.	.	.	.	1	10.0	1	2.9
**Blood cholesterol increased**	.	.	.	.	1	10.0	1	2.9
**Blood triglycerides increased**	.	.	.	.	2	20.0	2	5.9
**COVID-19 pneumonia**	.	.	.	.	1	10.0	1	2.9
**Epistaxis**	.	.	.	.	1	10.0	1	2.9
**Fibrin D dimer increased**	.	.	.	.	1	10.0	1	2.9
**Gamma-glutamyltransferase increased**	.	.	.	.	2	20.0	2	5.9
**Hyperglycemia**	.	.	.	.	1	10.0	1	2.9
**Interstitial lung disease**	.	.	.	.	1	10.0	1	2.9
**Low density lipoprotein increased**	.	.	.	.	1	10.0	1	2.9
**Nasal congestion**	.	.	.	.	1	10.0	1	2.9
**Serum ferritin increased**	.	.	.	.	2	20.0	2	5.9

N: number.

Supplementary Table 6. Six-minute walk test by severity of disease.

Categorical Endpoints (6MWT)			Mild		Moderate		Severe		Total	
			N	%	N	%	N	%	N	%
**Pre-TEST**	**Borg scale of dyspnea and fatigue at basal (1)**	**0**	5	100.0	16	84.2	10	100.0	31	91.2
		**1**	.	.	1	5.3	.	.	1	2.9
		**2**	.	.	2	10.5	.	.	2	5.9
**Post-TEST**	**Borg scale of dyspnea and fatigue after test (1)**	**0**	1	20.0	5	26.3	5	50.0	11	32.4
		**1**	2	40.0	3	15.8	1	10.0	6	17.6
		**1.5**	.	.	.	.	1	10.0	1	2.9
		**2**	.	.	5	26.3	1	10.0	6	17.6
		**2.5**	1	20.0	.	.	.	.	1	2.9
		**3**	.	.	3	15.8	1	10.0	4	11.8
		**4**	.	.	1	5.3	.	.	1	2.9
		**5**	.	.	1	5.3	.	.	1	2.9
		**6**	1	20.0	1	5.3	.	.	2	5.9
		**9**	.	.	.	.	1	10.0	1	2.9
**Results**	**Stopped or paused**	**No**	5	100.0	18	94.7	9	90.0	32	94.1
		**Yes**	.	.	1	5.3	1	10.0	2	5.9
	**Symptoms at the end of the exercise**	**None**	5	100.0	19	100.0	10	100.0	34	100.0

(1) 0 (None); 0.5 (Very, very slight), 1 (Very slight); 2 (Slight); 3 (Moderate); 4 (Somewhat severe); 5 (Severe); 6 (Severe); 7 (Very severe); 8 (Very severe); 9 (Very very severe); 10 (Maximal).

6MWT: 6-minute walk test.
